# Successful Percutaneous Coronary Intervention in a Patient With Absent Right Coronary Artery Ostium: A Case Report and Literature Review

**DOI:** 10.7759/cureus.59378

**Published:** 2024-04-30

**Authors:** Sahil Zaveri, Ezra Schrem, Kazim Aykent, Samy I McFarlane, Puneet Gandotra, Adam S Budzikowski

**Affiliations:** 1 Internal Medicine, State University of New York (SUNY) Downstate Health Sciences University, New York, USA; 2 Cardiovascular Research Program, Veterans Affairs (VA) New York Harbor Healthcare System, New York, USA; 3 Cardiology, State University of New York (SUNY) Downstate Health Sciences University, New York, USA; 4 Cardiology, South Shore University Hospital, Bay Shore, USA

**Keywords:** percutaneous coronary intervention, right coronary artery, coronary artery disease, cardiac catheterization, interventional cardiology

## Abstract

Coronary artery anomalies present unique interventional challenges, particularly when associated with significant coronary artery disease. This case report contributes to the limited literature on congenital coronary artery anomalies, emphasizing the need for tailored approaches to optimize patient care. We present a case of a 70-year-old male with refractory angina and a rare congenital coronary anomaly characterized by the absence of the right coronary artery ostium, necessitating reliance on the left coronary system for myocardial perfusion. Cardiac catheterization revealed mid-left anterior descending artery stenosis, prompting percutaneous coronary intervention. Despite the anatomical complexities encountered, the procedure was successfully performed. This case underscores the importance of meticulous diagnostic evaluation, advanced imaging techniques, and a multidisciplinary approach to managing patients with rare coronary anomalies. This report also emphasizes the unique diagnostic and therapeutic considerations by providing a comprehensive literature review and identifying areas for further research to advance treatment strategies and improve outcomes.

## Introduction

Coronary artery anomalies, though infrequent, represent a fascinating aspect of cardiovascular pathology, often presenting clinicians with diagnostic and therapeutic conundrums. Among these anomalies, the congenital absence of the right coronary artery (RCA) ostium is an exceptionally rare occurrence, characterized by the exclusive reliance of the right side of the heart on the left coronary system for myocardial perfusion [1,2]. Such anomalies challenge the conventional understanding of cardiac anatomy and underscore the intricacies of coronary circulation and its clinical implications [3,4]. We present a case of a 70-year-old male patient who underwent cardiac catheterization due to refractory angina. Coronary angiography revealed significant stenosis of the mid-left anterior descending artery (mLAD), coupled with the absence of a discernible RCA ostium.

## Case presentation

**Figure 1:**
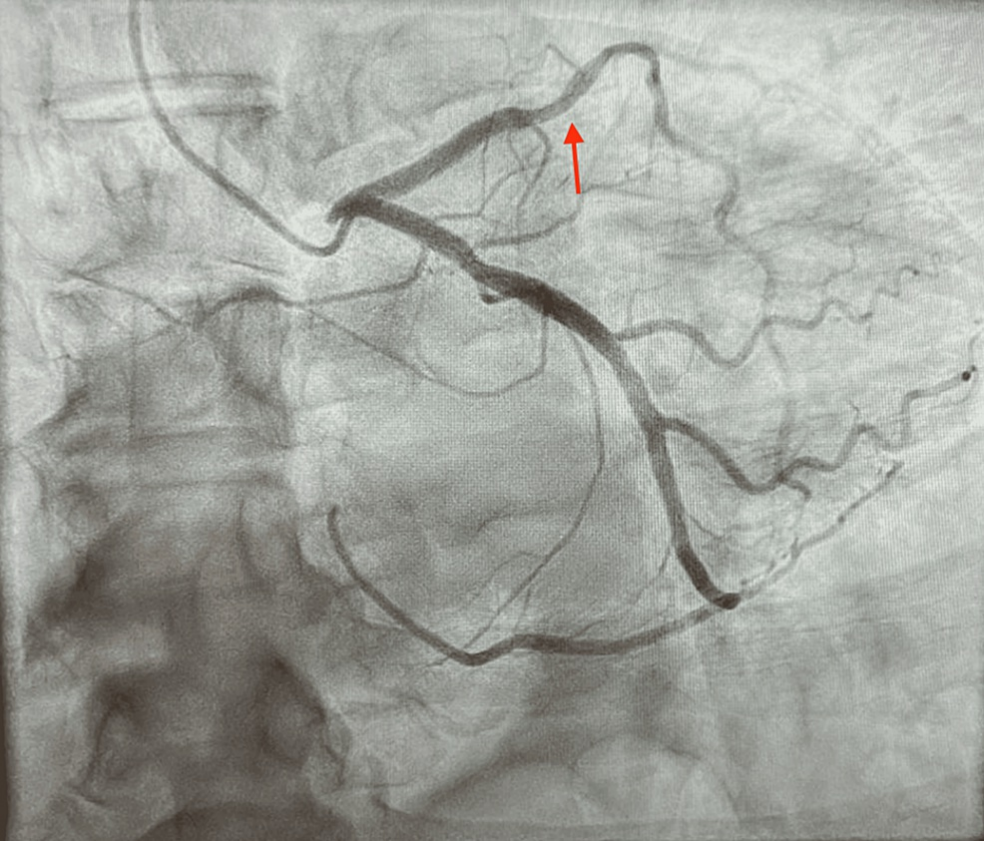
Selective left coronary angiogram in right anterior oblique caudal projection demonstrating severe stenosis in the mid-left anterior descending artery

**Figure 2:**
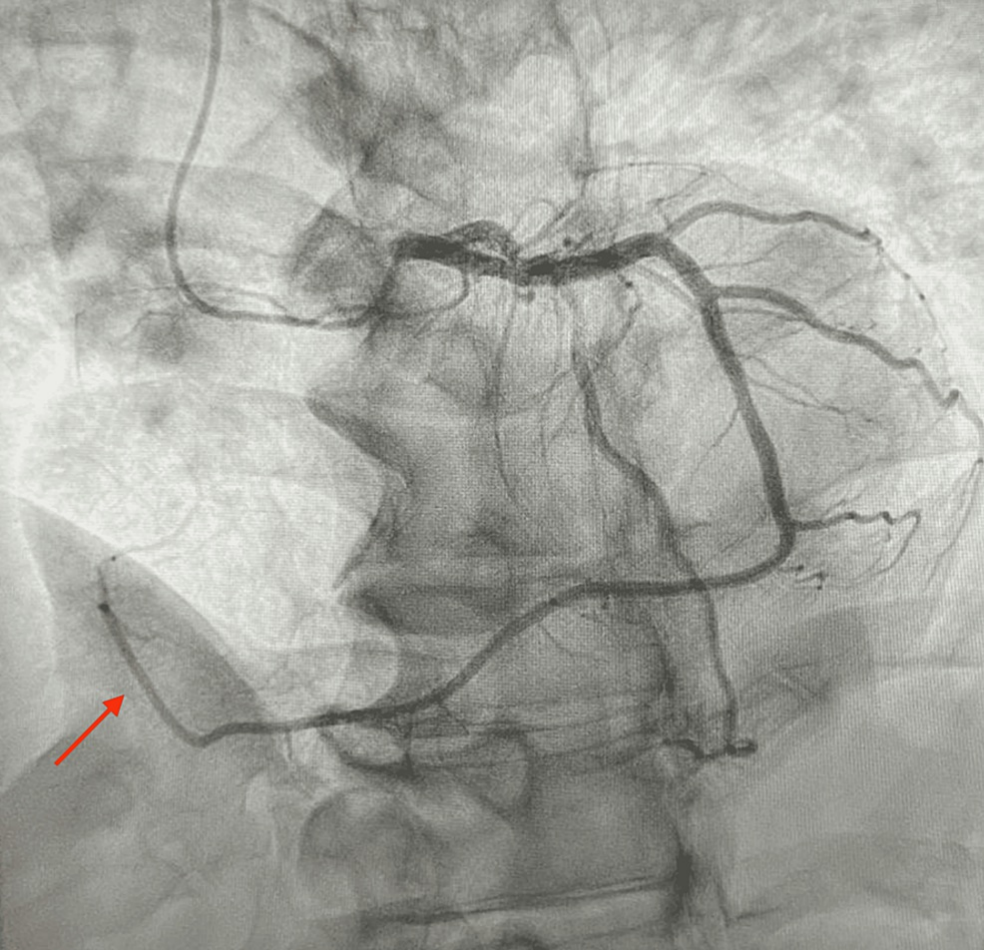
Selective left coronary angiogram in left anterior oblique cranial projection highlighting the congenital anomalous takeoff of the entire right coronary artery from the left circumflex artery

A 70-year-old male with a past medical history of hypertension, diabetes mellitus, hyperlipidemia, and former tobacco use presented with persistent angina refractory to medical therapy. Given his clinical history and the presence of a known 60% lesion in the mLAD observed during a previous left heart catheterization in 2021, he was scheduled for repeat elective catheterization to reevaluate his coronary anatomy. The procedure, performed via the right radial approach, revealed a single vessel coronary artery disease (CAD) with significant calcific stenosis of the mLAD at 75% (Figure 1). The initial unsuccessful engagement of the RCA prompted the operator to engage the left coronary system, which demonstrated the anomalous origin of the RCA from the left circumflex artery. This confirmed that the initial presumed unsuccessful attempts of RCA engagement were due to the absence of the RCA ostium, highlighting the unique anatomical variant in our patient (Figure 2).

**Figure 3:**
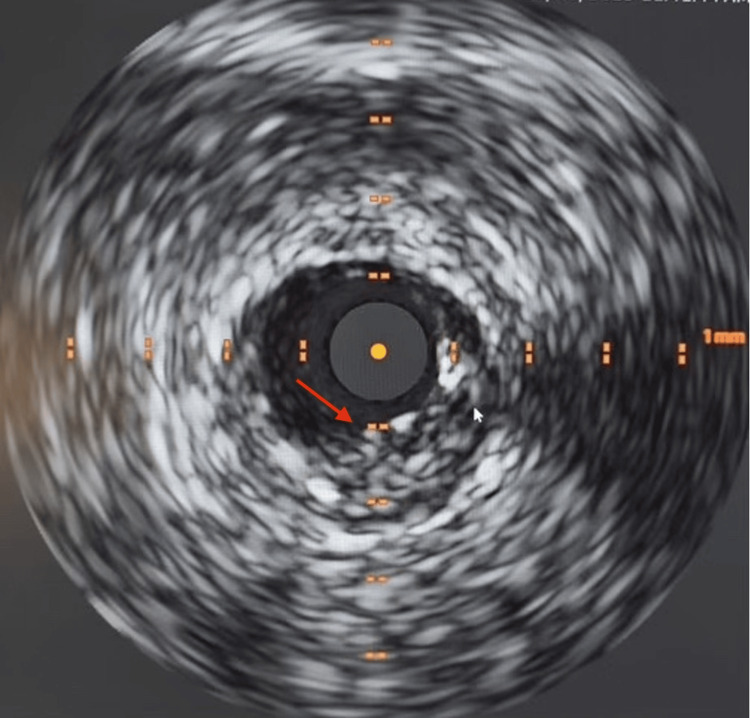
Intravascular ultrasound revealing severe mid-left anterior descending artery stenosis with bulky eccentric plaque burden

**Figure 4:**
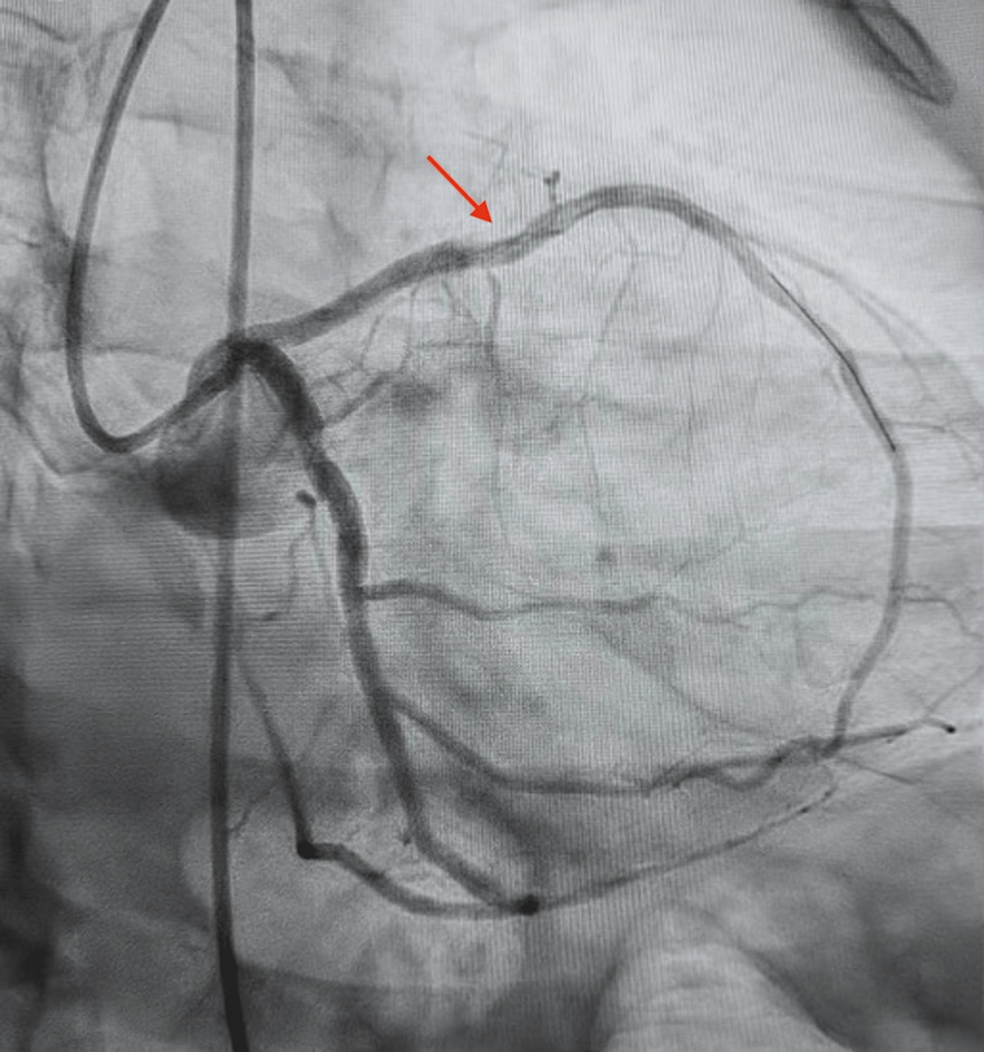
Post-percutaneous coronary intervention stent placement image showing complete resolution of the mid-left anterior descending artery lesion with no intracoronary complications

Following the diagnostic confirmation of mLAD severe stenosis with intravascular ultrasound and the absence of the RCA, the patient was deemed a candidate for percutaneous coronary intervention (PCI) to alleviate his symptomatic CAD (Figure 3). Despite initial concerns surrounding the anatomical complexity and potential risks associated with the single coronary ostium, PCI of the mLAD was performed successfully (Figure 4). The patient tolerated the procedure without any immediate complications and demonstrated continued hemodynamic stability post-procedure. Further follow-up will be conducted to monitor the patient's progress and manage his cardiovascular health effectively.

## Discussion

Our case report unveils a rare and unique coronary anomaly characterized by the congenital absence of the RCA ostium, necessitating reliance on the left coronary system for myocardial perfusion. This infrequent anatomical variation emphasizes the diverse complexity of coronary artery anomalies, posing significant technical challenges to PCI [3].

The successful completion of PCI in our patient underscores the advancing capabilities of interventional cardiology in managing complex CAD. While our case highlights procedural success, it is imperative to acknowledge the inherent risks associated with such interventions, including potential complications jeopardizing the entire coronary circulation [2]. Thus, meticulous pre-procedural planning, technical precision, and vigilant hemodynamic monitoring are pivotal in ensuring optimal outcomes in patients with rare coronary anomalies. 

Selecting and executing appropriate diagnostic imaging modalities is crucial in managing patients with complex congenital coronary anomalies, including the absence of the RCA ostium [5]. Traditional angiography, as opposed to coronary computed tomography angiography, may have limitations in identifying the anomalous origin of coronary arteries [6]. Villa et al. underscored the significance of coronary computed tomography angiography in identifying anomalous coronary origins, thereby facilitating treatment decisions [7]. The integration of advanced imaging modalities alongside traditional angiography is indispensable for accurate anatomical characterization and procedural optimization in patients with complex coronary artery anomalies [7].

While infrequent, similar cases of single coronary ostium anomalies have been sporadically documented in medical literature, emphasizing the diagnostic intricacies and therapeutic challenges associated with such anatomical variations [1]. Angelini et al. highlighted similar cases originating from the left coronary sinus, illustrating the diagnostic complexities posed by such anomalies [8]. Moreover, the successful outcomes of PCI in similar cases emphasize the significance of meticulous procedural planning and technical expertise in achieving favorable outcomes [4]. 

## Conclusions

We presented a rare case of congenital coronary anomaly characterized by the absence of the RCA ostium, highlighting the intricate nature of coronary artery variations and the challenges they pose in clinical management. Through meticulous diagnostic evaluation and successful PCI, we have demonstrated the adaptability of interventional cardiology in navigating complex coronary anatomies to achieve favorable outcomes. However, our case also emphasizes the need for a multidisciplinary approach, incorporating advanced imaging modalities and collaborative decision-making to optimize patient care. Further research, guided by evidence from published literature, is imperative to elucidate optimal diagnostic and therapeutic strategies for patients with rare coronary anomalies, ultimately improving clinical outcomes and enhancing the quality of care in this challenging subset of patients. This case serves as a valuable addition to the limited body of literature. It underscores the importance of continued efforts to advance our understanding of coronary artery anomalies and refine treatment algorithms to benefit patients with complex cardiovascular pathology.

## References

[1] Ahmed A, Assaf A, Mantha Y, Small D, Zughaib M (2021). A rare anatomical variant: congenital absence of the right coronary artery with left circumflex artery supplying the right coronary artery (RCA) territory. Am J Case Rep.

[2] Chen Z, Yan J, Han X (2020). Congenital absence of the right coronary artery with acute myocardial infarction: report of two cases and review of the literature. J Int Med Res.

[3] Cinelli M, Rahming H, Assaad M (2022). A curious case of an anomalous right coronary artery. Cardiol Res.

[4] Liu WC, Qi Q, Geng W, Tian X (2020). Percutaneous coronary intervention for congenital absence of the right coronary artery with acute myocardial infarction: a case report and literature review. Medicine.

[5] Elbadawi A, Baig B, Elgendy IY (2018). Single coronary artery anomaly: a case report and review of literature. Cardiol Ther.

[6] Yan GW, Bhetuwal A, Yang GQ (2018). Congenital absence of the right coronary artery: a case report and literature review. Medicine.

[7] Villa AD, Sammut E, Nair A, Rajani R, Bonamini R, Chiribiri A (2016). Coronary artery anomalies overview: the normal and the abnormal. World J Radiol.

[8] Angelini P, Velasco JA, Ott D, Khoshnevis GR (2003). Anomalous coronary artery arising from the opposite sinus: descriptive features and pathophysiologic mechanisms, as documented by intravascular ultrasonography. J Invasive Cardiol.

